# Doll therapy intervention for women with dementia living in nursing homes: a randomized single-blind controlled trial protocol

**DOI:** 10.1186/s13063-020-4050-8

**Published:** 2020-02-03

**Authors:** Roberta Vaccaro, Roberta Ballabio, Valentina Molteni, Laura Ceppi, Benedetta Ferrari, Marco Cantù, Daniele Zaccaria, Carla Vandoni, Rita Bianca Ardito, Mauro Adenzato, Barbara Poletti, Antonio Guaita, Rita Pezzati

**Affiliations:** 1 Golgi Cenci Foundation, Corso San Martino 10, 20081 Abbiategrasso, Italy; 2 Dipartimento di Economia Aziendale e Socio Sanitaria (SUPSI), Centro Competenza Anziani, Manno, Switzerland; 3 School of Cognitive Therapy, Como, Italy; 4 Dipartimento di Medicina di Laboratorio EOLAB, Ente Ospedaliero Cantonale, Bellinzona, Switzerland; 5 Department of Neuroscience ‘Rita Levi Montalcini’, University of Turin, Turin, Italy; 6 Department of Psychology, University of Turin, Turin, Italy; 7 Department of Neurology and Laboratory of Neuroscience, Istituto Auxologico Italiano, IRCCS, Milan, Italy

**Keywords:** Doll therapy, Dementia, Behaviour, Caregivers, Attachment

## Abstract

**Background:**

Doll therapy is a non-pharmacological intervention for people with dementia aimed to reduce distressing behaviours. Reliable results on the efficacy of Doll therapy for people with dementia are needed. The concept of attachment theorised by Bowlby has been proposed to explain the Doll therapy process, but it has not been proven to influence the response to doll presentation.

**Methods/design:**

This single-blind, randomised controlled trial will involve people with dementia living in nursing homes of the Canton Ticino (Switzerland). Participants will be randomised to one of two interventions: Doll Therapy Intervention or Sham Intervention with a non-anthropomorphic object, using a 1:1 allocation ratio. The two interventions will consist of 30 daily sessions lasting an hour at most, led by a trained nurse for an hour at most. We will enrol 64 participants per group, according to power analysis using an estimated medium effect size (*f* = 0.25), an alpha level of 0.05, and a power of 0.8. The primary goal is to test the efficacy of the Doll Therapy Intervention versus the Sham Intervention as the net change in the following measures from baseline to 30 days (blinded outcomes): the Neuropsychiatric Inventory-Nursing Home administered by a trained psychologist blinded to group assignment, the professional caregivers’ perceived stress scale of the Neuropsychiatric Inventory-Nursing Home, patients’ physiological indices of stress (salivary cortisol, blood pressure and heart rate) and interactive behaviours. The secondary goal is to assess the relationship between attachment styles of people with dementia (detected by means of the Adult Attachment Interview to the patients’ offspring) and their caregiving behaviours shown during the Doll Therapy Intervention.

**Discussion:**

This is the first single-blind, randomised controlled trial on the efficacy of Doll therapy for dementia and an explanatory model of the response of people with dementia to doll presentation.

**Trial registration:**

ClinicalTrials.gov, ID: NCT03224143. Retrospectively registered on 21 July 2017

## Background

Doll therapy (DT) is a non-pharmacological intervention to support people with dementia (PWD) and is recommended for the treatment of behavioural and psychological symptoms of dementia (BPSD) [1]. Previous literature provided some direction on how to use dolls as a nursing instrument [2–6]. Observational studies have shown the benefits of DT in reducing BPSD such as agitation, aggression and wandering [7, 8]. They have also shown the benefits of DTS in increasing communication between patients and caregivers due to the fact that dolls stimulate conversation on topics related to motherhood and caregiving [9, 10]. A significant reduction of antipsychotic drug dosage (e.g. haloperidol) has also been reported [11]. Furthermore, the benefits provided from DT fulfil the bioethics’ concepts of beneficence (facilitates the promotion of well-being) and respect for autonomy (PWD can exercise their right to engage with dolls if they wish) [12]. Despite promising results, available studies on the efficacy of dolls’ use are mainly pilot or exploratory studies [13–15]. Randomised controlled trials are needed in order to support the clinical efficacy of DT in managing BPSD and reducing professional caregivers’ perceived stress linked to BPSD. Moreover, empirical research is needed to identify best practice for DT interventions for PWD because there are differences among approaches to the practice being advocated. There are also difficulties in standardising the design and delivery of such complex interventions [16].

Another issue is the explanatory model of the DT process. Some authors have applied the concept of attachment theorised by John Bowlby to the ‘doll’ object [15, 17]. Attachment theory holds that the propensity of human beings to seek closeness and protection when feeling vulnerable or frightened is an expression of an innate motivational system. This system activates attachment behaviours directed to achieve proximity and protection from another person (i.e. crying, calling, approaching and holding). The attachment behaviours persist during the entire life cycle, ‘from the cradle to the grave’ [18], and are linked to three types of mental states: secure, insecure and unresolved [19]. This motivational system is particularly relevant for PWD, since dementia often exposes them to feelings of personal vulnerability [20, 21]. The observation of DT interventions revealed that PWD recognised the doll as a real baby and replaced the requests for care and protection (through attachment behaviours, i.e. vocalisations, gestures and tears) with caregiving behaviours (i.e. reassuring and cradling the doll and restoring a sense of calm and peacefulness). The attachment theory could frame the therapeutic function of the doll for PWD in the advanced stages. Such an explanatory model of the DT process has been proposed in a previous study [15], but more robust empirical research is needed in order to confirm it.

In the present single-blind, randomised controlled trial, the first hypothesis regards the 30-day efficacy of the DT on BPSD, professional caregivers’ perceived stress linked to BPSD, patients’ physiological indices of stress, and interactive behaviours. The second hypothesis is that the past attachment styles, embedded during one’s life cycle, are stable even in the advanced stages of dementia. It is also hypothesised that the caregiving behaviours of PWD shown during the doll presentation are linked to their attachment styles.

## Methods/design

### Primary goal

The primary goal of the present study is the 30-day efficacy of the Doll Therapy Intervention (DTI) versus a Sham Intervention (SI) on BPSD, professional caregivers’ perceived stress, patients’ physiological indices of stress, and interactive behaviours of PWD living in a nursing home. In particular, we expect an improvement in terms of a decrease in the Italian version of the Neuropsychiatric Inventory-Nursing Home total score (NPI-NH) [22, 23]. We also expect an improvement of professional caregivers’ perceived stress linked to BPSD of PWD in terms of a decrease in the NPI-NH Distress (NPI-NH-D) total score. Furthermore, we expect an improvement of the patients’ physiological indices of stress linked to their BPSD in terms of a decrease in the following parameters: salivary cortisol, blood pressure (systolic and diastolic) and heart rate, which are well-known biomarkers of acute stress. Finally, we expect an improvement of the patients’ interactive behaviours towards the object (doll) in terms of an increase in the number and duration of exploratory and caregiving behaviours.

### Secondary goal

The secondary goal is the confirmation of the hypothesised stability of the past attachment style of the PWD (provided by the Adult Attachment Interview (AAI) [24] administered to the patients’ offspring (family caregivers), even in the advanced stages of dementia. The current attachment style of the PWD is provided by the observations of the patient during a situation of meeting-separation from their own family caregiver. These evaluations will be conducted, after conclusion of interventions, among a sub-group of participants in the experimental condition. We expect to find an association between the past and the current attachment style of the PWD, categorised as secure, insecure, and unresolved. Furthermore, the relationship between patients’ attachment styles and the response to DTI in terms of caregiving behaviours towards the doll (e.g. caressing the object, talking to it and smiling) will be investigated. We expect that PWD with a previous secure attachment style will accept the doll and will show more caregiving behaviours compared to PWD with insecure attachment style, while these latter will show more rejection, avoidance or excessive caregiving reactions towards the doll. We also expect that PWD with unresolved attachment style will show inconsistent responses to the doll presentation, with approaching and rejecting behaviours.

### Ethical considerations

The study procedures are in accordance with the principles outlined in the Declaration of Helsinki of 1964 and the following amendments. The study protocol was submitted to, and approved by, the Swiss Ethics Committees on Research Involving Humans (Ref. no. CE3140 BASEC2016–01992). The recruitment phase is ongoing. Briefly, during recruitment of the participants we check the presence of the aforementioned inclusion and exclusion criteria in order to establish the eligibility of each patient. Then, the patients or their legal representative (families) will be asked to sign a written informed consent for the use of their personal data and will receive a concise and comprehensive briefing explaining the research objectives and methods of data processing.

### Study design

**Fig. 1:**
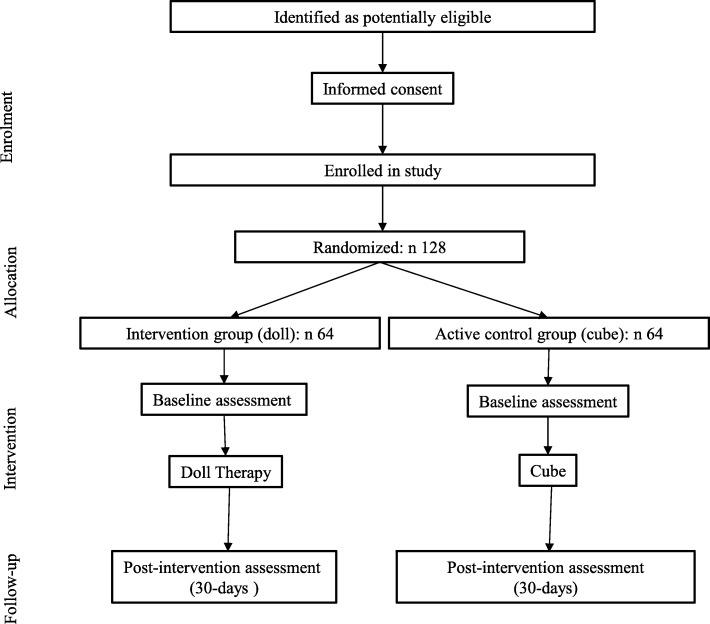
Trial flow chart

This study is a single-blind, randomised controlled trial with two parallel arms, conceived to assess the 30-day efficacy of the DTI compared to SI with a non-anthropomorphic object in PWD living in nursing homes (Fig. 1). The study is conducted and reported in accordance with the Consolidated Standards of Reporting Trials (CONSORT) guidelines for non-pharmacologic treatments [25] and with the SPIRIT (Standard protocol Items: Recommendations for Interventional Trials) guidelines (see Additional file 1). Principles of the Medical Research Council (MRC) Framework have inspired us in order to develop the DTI, to standardise the experimental and SIs, to set up procedures for monitoring delivery of the intervention, to evaluate the interventions and describe in depth all the procedures to enable replication studies [16]. The study is part of a larger project focussing on DT dissemination in Canton Ticino (Switzerland) through team training and supervision and guidelines‘ definition that led to the establishment of the Ticino Doll Therapy Group with support of the Canton Office for Elderly and Home Care. The phases of the project are as follows:

1. A first phase aimed to inform all nursing home operators and patients’ relatives about the establishment of a training programme on DT that they can join if interested
2. An ad hoc training phase addressed to professional operators. Nursing assistants are trained to use dolls and to record responses through appropriate daily grids
3. The research phase, described below, that will take place at the end of the informative and training phase

### Participants and setting

Participants will be PWD living in 22 nursing homes of the Canton Ticino area, Switzerland. Participants will meet the following inclusion criteria: presence of moderate to severe dementia, assessed using the Global Deterioration Scale (GDS 4–7) [26]; presence of at least one BPSD in addition to depression or apathy assessed with the NPI-NH; female gender; admission in the nursing home at least 3 months before; and no previous DT exposure. The exclusion criteria were the following: male gender; presence of comorbid mental disorders, such as major depression, bipolar disorders and schizophrenia; inability to sit comfortably on a chair or limitations in mobility of the arms; or presence of acute clinical conditions interfering with the participation to the study.

### Sample size

To our knowledge, at the start of the study on 1 February 2017, no previous study had measured the efficacy of DT using the NPI-NH score. Therefore, a referral upon existing data could not be used in the power calculation. The power analysis was conducted using an estimated medium effect size (*f* = 0.25), an alpha level of 0.05, and a power of 0.8. According to statistical computing, a sample size of *n* = 128 is required, with 64 participants per group, for an analysis of covariance that includes the NPI-NH baseline score as a covariate. We planned an increase in sample size of 13 participants, because the expected drop-out rate is 10% due to possible acute clinical conditions interfering with the participation in the study, or death. In case of a refusal of the object (doll or cube), another participant will be enrolled as a replacement. Participants who underwent less than 60% of sessions will be excluded from analysis. The power calculation was performed with the programme G*Power 3.1 [27].

### Randomisation and blinding

Participants will be assigned using a 1:1 concealed randomisation. The block randomisation will be computer based [28] and performed by an independent statistician. The statistician will generate the randomisation sequence and forward it to the study coordinator who will receive the randomisation for the allocation of the patients to either group A or B (block size: 2 × 2 = 4). To ensure blinding, the psychologist who administers the NPI-NH is not aware of the arm to which the participants belong. Conversely, the enrolled participants and the nurse are not be blinded with respect of the objects provided.

### Description of interventions

**Fig. 2:**
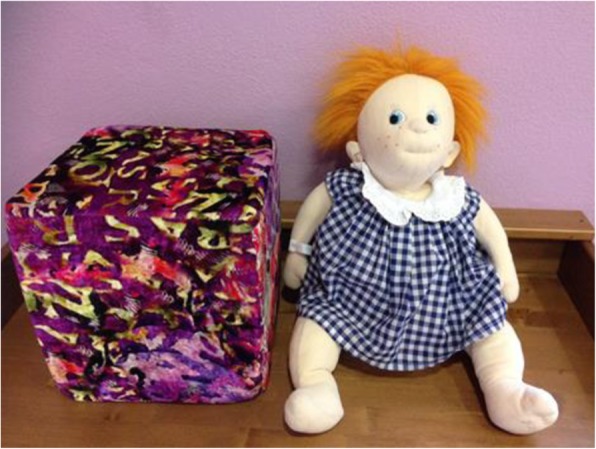
The doll and the soft cube

Participants will be randomly assigned to an experimental DTI group or to an active control group with SI. They will start the intervention within a week after randomisation. We chose to adopt an active control group instead of a no-treatment one to ensure comparability of experimental and control participants with respect to the efficacy of the intervention. Each participant will undergo daily sessions of DTI or SI*,* lasting an hour at most, led by a trained professional caregiver. Although the experimental phase of the study will be over, participants involved in DTI will continue to receive the doll, while participants involved in SI will start DT. The DTI involves the presentation of a doll produced by a Swedish brand and conceived for DT use. It is designed to recreate the sensation of touching, looking and holding a child in the arms. The SI group will attend similar daily sessions, but in place of the doll, it will be presented with a non-anthropomorphic object (i.e. a soft foam-rubber cube covered with a coloured and velvety textile) (Fig. 2).

The first and 30th sessions of all participants will be videotaped by a trained psychologist as described below.

All sessions (DTI and SI) will take place inside the residential complex in a room known to the patient, with a bed and a chair on which the participant will sit; the only people in the room will be a nurse and the researcher who handles video-recording. The latter will never interact with patients and will stand behind a closet outside their field of view.

The object (doll or cube) presentation procedure will be structured in five standard steps as follows:

1. The nurse will accompany the patient in the room and the patient will take a seat
2. The nurse will then leave the room and return with the doll or the cube. The nurse will put the doll or the cube in front of the patient and will say ‘Good morning Mrs....look.’ The nurse will gaze at the patient. The tone of voice will be quiet. The doll or the cube will be shown in the same way: they will be held with both arms in front of the patient and away from the operator’s body. If the patient will not take the doll or the cube at the first attempt, the nurse will sit down in front of her holding the object in the arms and will wait for 2 mins. The nurse will invite the PWD again by saying ‘Take it’ and ‘It is for you’. If the patient will not take the object after the second request, the nurse will not insist, she will leave and say: ‘I have to go, goodbye Mrs....’. If the object is taken, the nurse will not comment in any way and will not interact with the patient
3. She will leave the patient and say ‘I have to go, goodbye Mrs....’. During this procedure the gaze will always be upon the patient
4. Interaction with the object lasts 3 min starting from the moment when the nurse leaves the room. This phase is interrupted if the patient drops the object before the time limit
5. The nurse returns to the room and takes back the object

This procedure is based on the pilot study conducted by Pezzati et al. (2014) on ten residents of a special care unit for Alzheimer’s disease. The study has provided data supporting the feasibility and utility of the DTI in promoting well-being in patients with an advanced stage of dementia. The procedure is structured with the goal of recreating a situation of separation from a known figure and interaction with the environment in order to partially recreate the prototypical phases of the ‘strange situation’ [19]. The procedure has been simplified and adapted in order to make it administrable to institutionalised PWD and to highlight interactions with the offered items. All videotaped sessions will be analysed by the same evaluator, a trained psychologist who will fill the observational grids specifically developed for this study (see Tables 1 and 2). All participants will be assessed on the first day and the 30th day in the two groups (DTI and SI).

**Table 1:** Observational grid for caregiving behaviours of the person with dementia (PWD) during the object presentation (doll or cube) (fulfill after the 1st and the 30th session looking at the videotape)

Patient ID:	Date:	1st session:	30th session:
*Doll Therapy Intervention*:	*Sham Intervention (cube)*:		
	Presence	Absence	
1.Object presentation			
*1.a.Gaze direction*:			
Toward the nurse			
Toward the object			
Between the nurse and the object			
Immediately toward elsewhere			
Between the nurse and the object, then toward elsewhere			
Other			
*1.b.Responses to the object presentation*:			
Acceptance			
Refusal			
Avoidance of the contact with the object			
Talking			
Asking the nurse for a meaning of the situation			
2. Separation from the nurse			
*2.a Responses to the separation from the nurse*:			
Acceptance			
Complain			
Worry			
Lack of interest			
3. Behaviours during the interaction with the object			
*3.a. Exploration behaviours*:			
Exploration behaviours			
Duration of exploration behaviours (seconds)			
Types of exploration behaviours			
Touching			
Smelling			
Looking at the object			
Moving the object around			
Relocating the object			
Other			
3*.b. Caregiving behaviours*:			
Caregiving behaviours			
Duration of caregiving behaviours (seconds)			
Types of caregiving behaviours			
Tightening grip on the object			
Caressing			
Cradling			
Talking			
Smiling			
Playing			
Other			
4.Separation from the object			
Putting the object aside			
After how long does the patient puts the object aside (seconds)			
Looking for the nurse			
Calling the nurse			
Staying seated			

*PWD* people with dementia

**Table 2:** Observational grid for the situation of patient‘separation from the child (current attachment style of the person with dementia (PWD))

Patient ID:	Date:	
*Doll Therapy Intervention:*	*Sham Intervention (cube):*	
	Presence	Absence
*1. First stage: interaction between the patient and her child*		
*1.a. Gaze direction:*		
Toward the child		
Avoidant of the child		
Other		
*1.b. Responses to the interaction with the child:*		
Smiling		
Crying		
Seeking closeness		
Wandering		
Moving away from the child		
*2. Second stage: separation from the child*		
*2.a. Responses to the separation from the child:*		
Acceptance		
Complaining		
Worrying		
Lack of interest		
*3. Third stage: behaviours during the absence of the child*		
Crying		
Looking for the child		
Lack of interest		
Wandering		
Turning toward the stranger (the researcher)		
*4. Fourth stage: behaviours during the reunion with the child*		
*4.a. Gaze direction:*		
Toward the child		
Avoidant of the child		
Between the child and the room		
Other		
*4.b. Responses to the presence of the child:*		
Smiling		
Crying		
Screaming		
Seeking closeness		
Wandering		
Moving away from the child		
*NOTES:*		

## Measurements

### Primary outcomes

#### Behavioural outcome

The primary outcome is the decrease of the patient’s BPSD measured as the net change in the NPI-NH total score from baseline to post intervention. We expect a significant difference in NPI-NH total scores between the experimental and active control groups. The NPI-NH has been specifically designed for interviewing professional care facility staff; it has been validated in different countries, and the Italian version of the scale has demonstrated good psychometric properties [22]. It will be administered to the staff by a trained blinded psychologist. The time frame of that evaluation is from baseline (T0) to 30 days after the beginning of the intervention (T2).

#### Professional caregiver distress outcome

A decrease of the professional caregiver distress ratings related to the patient’s BPSD is also expected. It will be measured as the net change in the NPI-NH-D total score from baseline to post intervention [22, 23]. We expect a significant difference in NPI-NH-D total scores between the experimental and active control groups. It will be administered to the staff by a trained blinded psychologist. The time frame of that evaluation is from baseline (T0) to 30 days after the beginning of the intervention (T2).

#### Physiological outcomes

We have planned to monitor three physiological outcomes: blood pressure (systolic and diastolic), heart rate and salivary cortisol level. Blood pressure (systolic and diastolic) and heart rate are biomarkers of acute stress involved in the response to physical and psychosocial stressors. Salivary cortisol level is a biomarker of stress, and its secretion is the final product of the activation of stress-response mechanisms; specifically, the hypothalamic-pituitary-adrenal axis [29]. Changes in its secretion rate were reported during acute responses to stress in the context of illnesses and in cognitive impairment [30], in mood disorders [31] and in individuals with different adult attachment styles [32]. Professional caregivers will collect the saliva samples, blood pressure (systolic and diastolic) and heart rate readings of each participant in both the experimental and control groups, immediately before the treatment and 15 min after the end of treatment at the first and the 30th session of DTI or SI. We expect a significant difference between the experimental and the control group in blood pressure levels and heart rate from the first to the 30th session. A ratio between pre-treatment versus ‘after 15 min’ will be calculated for the first and 30th sessions. We expect a significant difference in the concentration of salivary cortisol between the experimental and control groups from the first to the 30th sessions.

#### Interaction with the object outcome

We expect an increase of the interaction with the object (doll or cube), as exploration and caregiving behaviours during the object presentation. These measures will be recorded at the first and the 30th sessions by a trained psychologist who will fill an observational grid. It is a non-validated instrument developed for the aims of the present study that includes four areas related to different kinds of behavioural responses in a dichotomous way (behaviour: present/absent; see Table 1): object presentation, separation from the nurse, behaviours during the interaction with the object and separation from the object. We expect a significant difference in frequency of the interactions with the object from the first to the 30th session between the experimental and control groups.

### Secondary outcomes

#### Confirmation of stability of attachment style

Assessment of premorbid attachment style of PWD (Adult Attachment Interview (AAI)).

We have planned to study 30 patient-offspring couples (about 47% of the participants in the experimental group). When offspring who regularly visit the patient have accepted the interview, the AAI will be administered to a son or daughter of a patient among those in the DTI group. The AAI is a semi-structured interview designed to evaluate attachment styles in adult populations and allows us to identify three different categories of mental states related to the attachment style of the son or daughter of the PWD: free, dismissing/preoccupied, or unresolved. Free adults have positive internal representations about past relationships with their parents and feel free to explore their infant experience. Dismissing or preoccupied adults (categorised as insecure attachment) fail in telling coherent attachment stories about their parents; they are inclined to idealise or denigrate the role of their parents in the past. Unresolved adults show problems with past mourning experiences and unresolved trauma occurring during infancy. Since a correspondence of 75% between the individual’s attachment and that of their children has been reported [33], we can hypothesise that the AAI is a useful instrument to infer the premorbid attachment style of PWD. This semi-structured interview will be administered in the nursing home and will be video-recorded. The coding of the answers will be carried out by an external trained and authorised psychologist.

#### Assessment of current attachment style of the PWD

After the interview, family caregivers will be invited to visit their relative. They will meet in a room of the nursing home and the task will be based to some extent upon ‘Ainsworth’s strange situation’, an experimental research setting originally conceived to observe young children. This situation will aim to facilitate exploration of possible attachment behaviours under different conditions. The visit will last about 15 min. There will be four distinctive stages:

1. The patient is together with the researcher, a trained psychologist in the role of stranger in a room. The family caregiver meets the patient. It is an unexpected visit
2. The family caregiver indicates, by talking and/or gesturing, that they must leave and suddenly ends the visit. This creates a potentially threatening moment for the PWD
3. The patient is once again in the room with the stranger
4. The family caregiver comes back to the room

The whole visit will be video-recorded. The occurrence of attachment behaviours will be determined afterwards by interval sampling. The presence or absence of attachment behaviours will be registered on an observational grid, a non-validated instrument developed for the present study (see Table 2).

We expect the stability of the attachment style of the PWD in term of a correlation between the premorbid attachment style and the current one. We also expect a correlation between the patients’ attachment styles and their response to DTI.

### Other variables

Other variables will be collected for the purpose of the present study, as follows: age of the PWD at the time of baseline; baseline Mini Mental State Examination (MMSE) score [34]; total number and relative dosages of psychopharmacological therapies for behavioural and psychiatric symptoms; presence of physical restraints; number of children and monthly visits to the PWD; age and gender of the offspring of PWD. A summary of the assessment performed at each time of the study is reported in Table 3.

**Table 3:**
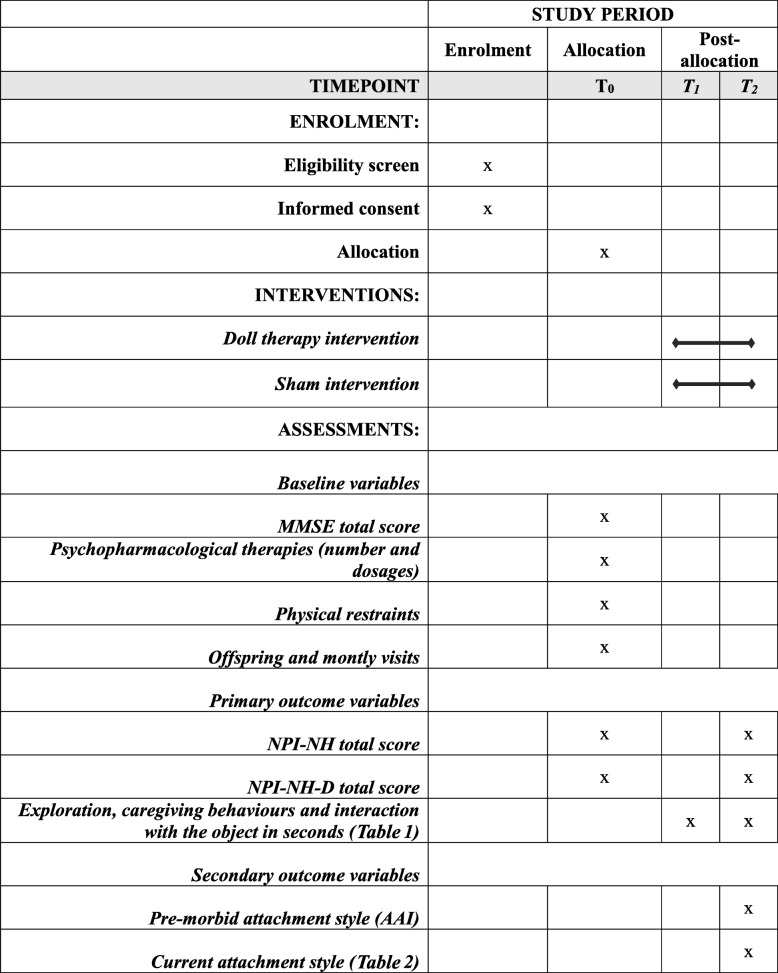
Content for the schedule of enrolment, interventions and assessments*

*MMSE* Mini Mental State Examination*, NPI-NH* Nursing Home version of Neuropsychiatric Inventory, *NPI-NH-D* Nursing Home version of NPI Distress, *AAI* Adult Attachment Interview

### Statistical analyses

As a first step, a post hoc evaluation by descriptive statistical comparisons between the intervention and the control groups in age and baseline NPI-NH will be conducted to assess the effectiveness of the randomised assignment. To evaluate associations between biological parameters (decreased, influent, or increased) of each patient at the baseline versus the post intervention, the chi-square test or Fisher’s exact test will be performed. Primary outcomes will be tested performing an analysis of covariance (ANCOVA) of these measurements with the experimental group as the factor baseline NPI-NH and total number and posology of psychopharmacological therapies as the covariates. In order to evaluate how much variance in NPI-NH net change can be explained by groups (experimental versus control groups) after controlling for baseline NPI-NH and biological parameters, a multiple regression analysis will be performed.

Treatment efficacy will be analysed by a per-protocol analysis (PPA) that excludes all drop-outs. Differences will be considered statistically significant when *p* values are lower than 0.05.

All analyses will be conducted using Stata version 13 (Stata Corp., College Station, TX, USA).

### Data management

Data transcription and data entry will be under responsibility of the Dipartimento di Economia Aziendale e Socio Sanitaria (SUPSI), and both will be entrusted to the trained researchers. Data ownership will remain with SUPSI which signed a contract of availability to their use for the purposes of research, dissemination of results and their publication. All visual and paper material (questionnaires, consents, etc.) will be stored at SUPSI in closets and filing cabinets fitted with keys. Information recorded on paper will be promptly transferred in a specifically designed password-protected electronic archive or database; the access to the personal computer containing the electronic implementation will be protected by an access key. In the database, each participant will be associated with an identification code without identity record, which will be stored separately and protected by a password known only to the research manager or her delegate. The code will constitute the key linkage between participants’ identity record and the information obtained during the DT study.

## Discussion

We report here the rationale and the design of the DT intervention for people with dementia living in nursing homes. The plausible mechanism that would explain the efficacy of DTI in reducing BPSD is related to the personhood of care practices for PWD that occurs via the engagement of the patient in meaningful activity. By using a situation of recognition of the doll as a real baby, challenging behaviours (viewed in our perspective as requests for care and protection, i.e. attachment behaviours) are replaced with caregiving behaviours. As occurs when PWD received proximity, similarly the re-activation of innate behaviours of care give them feelings of security. In our study, participants are in a stage of disease in which relationships become difficult and painful due to the difficulty the nurses and patients themselves encounter in reciprocal interpersonal and emotional tuning. The situation of reassurance offered by DT could promote the activation of the system of caregiving and of exploration. This could support an adaptation to the illness. Ultimately the experience of DT may promote the patient’s well-being [15].

The difficulty of professional caregivers at managing behavioural disturbances causes their perceived stress, as negative feelings and exhaustion, linked to taking care of the PWD every day [35, 36]. In the present protocol, the practices adopted during DTI with the aim of personhood of care enhance professional caregivers’ roles. They improve the patient’s observation, adopt a shared language and promote communication exchange about the PWD. Professional caregivers are more engaged in thinking about patients’ characteristics and in looking for new solutions to their behavioural problems. Via this process, we expect a reduction of their level of stress.

The attachment styles are recognised as being persistent during the ageing phases as demonstrated by previous studies based on the AAI, but the PWD cannot be interviewed. To date, there are no studies which have overcome this limitation. Moreover, the attachment style, experienced with the attachment figure since birth, may change during the life cycle. The experiences with other significant and the new adaptations to the demands posed by the various stages of life exert their influence acting as corrective. It is possible that, in the advanced stages of dementia, these adaptations could be lost due to the deterioration of capability of access to the meaning of emotional states. The comprehension of the attachment style of the PWD is of key importance, so that caregivers can interpret and respond to specific modalities of the PWD to ask for closeness and protection [37].

A limitation of this study is the exclusion of men. This is because women are 71.6% of patients with dementia living in nursing homes of the Canton Ticino, as reported by the Department of Health and Sociality of the Canton Ticino (source: Statistica intra-muros, SOMED, Dipartimento della sanità e della socialità, Unità statistiche sanitarie, Bellinzona). The authors have chosen to exclude men in order to have the most homogeneous sample in the time frame of the study, but further research is needed in order to demonstrate the feasibility of DT in men. The need for attachment and the ability to provide assistance are linked to the innate drive to give security and comfort to an individual who presents physiognomic characteristics of fragility and vulnerability (small nose, high forehead, soft skin). These characteristics activate caregiving behaviours and oxytocin increase, supporting the caregiving activity: this is not about gender but about characteristics belonging to all humankind [38]. Physiological parameters will always be monitored during the interventions (first or 30th session) both for the experimental (doll) and active control groups (cube), but we will not collect the basal stress levels prior to any interference of the intervention (cube or doll).

Another limitation of the present study is that we have not planned a cost-effectiveness evaluation: although there is some chance of benefit at low cost, data limitations preclude a definitive analysis. In summary, this will be the first study on DT efficacy for the PWD living in nursing homes adopting a single-blind, randomised controlled design. We expect that it will provide generalisable data in order to identify best practice for non-pharmacological interventions for PWD and to overcome differences among approaches to the practice being advocated.

## Trial status

This study is currently in the process of recruiting participants. The study was registered on 21 July 2017 (protocol version number: 1), it started on February 2017 and it is expected to finish in February 2020.

## Supplementary information

**Additional file 1.** Standard Protocol Items: Recommendations for Interventional Trials (SPIRIT) 2013 Checklist: recommended items to address in a clinical trial protocol and related documents.

## Data Availability

Not applicable

## Abbreviations

AAI: Adult Attachment Interview
ANCOVA: Analysis of covariance
BPSD: Behavioural and psychological symptoms of dementia
CONSORT: Consolidated Standards of Reporting Trials
DT: Doll therapy
DTI: Doll Therapy Intervention
GDS: Global Deterioration Scale
MMSE: Mini Mental State Examination
NPI-NH: Nursing Home version of Neuropsychiatric Inventory
NPI-NH-D: Nursing Home version of NPI Distress
PPA: Per-protocol analysis
PWD: People with dementia
SI: Sham Intervention
